# Supplementary material for: Are carbon emissions trading and green financial instruments synergistic? -Comprehensive quantitative research based on content analysis
Source: PLoS One. 2024 Mar 7;19(3):e0298601. doi: 10.1371/journal.pone.0298601 (PMC10919601; doi:10.1371/journal.pone.0298601)
Supplement: S1 Data — (ZIP) [file pone.0298601.s001.zip › Data/地级市Z_F工作报告.zip!/║╙─╧/╫ñ┬φ╡Ω╩╨/└┤╫╘╣1⁄2╓┌║┼í╛┬φ┐╦╩2╛▌═°í┐_2011.docx]

各位代表

现在，我代表市人民政府，向大会作工作报告，请予审议，并请各位政协委员提出意见。

折叠编辑本段2010年及"十一五"工作回顾

折叠国民经济保持平稳较快增长

面对国际金融危机影响仍在继续的严峻形势，我们紧紧围绕发展主题，采取有效措施，认真解决经济运行中出现的矛盾和问题，保持了经济平稳较快增长的良好势头。初步核算，全市生产总值达到1055.45亿元，增长11.6%。财政总收入61.9亿元，增长20.9%，其中地方财政一般预算收入36.4亿元、支出172.67亿元，分别增长24.3%、20.3%。积极扩大投资。全市112个重点建设项目完成投资137亿元，占年度计划的113.5%。国电驻马店热电2×33万千瓦供热机组扩建、顺达化工年产36万吨醋酸技改等重大工业项目快速推进，豫龙同力日产4500吨水泥熟料生产线、220千伏确山输变电工程竣工投产。焦桐高速泌阳段建成通车，石武客专驻马店段、化新高速新蔡段超额完成年度投资计划。城镇固定资产投资496.74亿元，增长21.5%。着力扩大消费。认真落实国家鼓励扩大消费政策，发放家电下乡和汽车、摩托车下乡补贴3.81亿元;商品房销售面积428.26万平方米，销售额达74.74亿元，增长43.7%。社会消费品零售总额376.67亿元，增长18.4%。努力扩大对外贸易。积极培育对外贸易主体，调整出口商品结构，全年完成进出口总额2.32亿美元，其中出口1.78亿美元，增长38%;进口5377万美元，增长408%。深入开展服务企业活动。市、县确定重点服务企业806家，建立健全服务企业长效机制，加强煤、电、油、气、运等要素保障，积极引导金融机构加大信贷投放，有力地支持了工业发展。全部工业增加值达到393亿元，其中规模以上工业增加值279.84亿元，增长21.5%;利润81.46亿元，增长44.1%。

折叠经济结构调整取得新进展

坚持在发展中调整、在调整中提升，加快发展方式转变，经济发展的质量效益明显提高。产业结构得到优化。传统产业技术改造步伐加快，高端优势产业健康发展，食品、医药、建材、制造、能源和煤化工等支柱产业规模继续壮大，增加值比重达到63.1%。商贸、餐饮等传统服务业持续提升，文化、旅游、物流等服务业快速发展，第三产业增加值达到323.5亿元，增长12.6%。二、三产业比重达到72.5%。产业集聚区建设步伐加快。大力推进基础设施和公共服务平台建设，12个产业集聚区全部通过省级评审，累计实施工业项目476个，一批特色产业集群初步形成。全年产业集聚区完成投资311.9亿元，实现主营业务收入629亿元。自主创新能力增强。认真实施质量兴市战略，加强自主创新体系建设，38家企业工程技术研究中心带动企业实现销售收入200亿元、实现税金13亿元。全市申报认定省级工程技术研究中心4个，新建市级工程技术研究中心16个，专利申请量达540件。节能减排和生态环境建设扎实推进。万元GDP能耗下降4.72%，化学需氧量、二氧化硫两个主要污染物排放量实现双降，圆满完成省定"十一五"减排目标。深入推进林业生态市建设，完成造林23.46万亩，9个县区实现了林业生态县创建目标。

折叠"三农"工作进一步加强

各项强农惠农政策全面落实，投入继续加大，"三农"支出达24.3亿元。粮食生产能力稳步提升。全年共落实各类惠农补贴17亿元，争取上级水利投资14.12亿元，改造中低产田13.6万亩，新增、改善有效灌溉面积37万亩，粮食生产再获丰收，总产达133.4亿斤，连续7年实现丰收。农业结构调整扎实推进。高效农业规模不断扩大，优质专用小麦种植面积553万亩，总产26.15亿公斤。现代畜牧业快速发展，新建较大规模的畜禽养殖场区356个，肉、蛋、奶总产量分别增长5%、8%和10%。花木、蔬菜、食用菌、花生等特色农业稳步发展。市级以上重点龙头企业达到276家。农村生产条件继续改善。全年新建、改建农村公路325公里、通村公路179公里。新建农村户用沼气2万户，解决了农村50.5万人的安全饮水问题，11.54万农村人口实现脱贫，完成了3个电气化县、61个电气化乡镇、662个电气化村的建设任务。53个省、市级新型农村住宅社区试点开工建设。农机总动力1326.9万千瓦，农作物耕种收机械化率78.42%。

折叠城镇化进程加快

大力推进城镇建设与管理，全市城镇化率达到32%，提高2.5个百分点。编制了"三区两县一体化"总体规划、中心城区城市空间发展战略规划，启动了职教园区、产业集聚区控制性详规编制工作，中心城区控规覆盖率达到76%，完成了九县一区村镇体系规划和145个乡镇总体规划。坚持以"四城联创"为载体，大力实施畅通、环卫和绿化、美化、亮化工程，汝河大道东段、驿城大道南段、练江河中段综合治理等7个项目顺利完成，铜山大道、中原大道等12个项目扎实推进，新建改造了一批游园广场、环卫以及供排水等公共设施，城市建管水平明显提高。县城和小城镇建设快速推进，城镇功能逐步完善。全年城市基础设施投资63亿元，增长22.1%，其中中心城区完成投资21亿元，是上年的3.7倍;建成区面积达60平方公里，较上年增加10平方公里。同时，积极破解户籍、就业、住房和社会保障等体制机制障碍，认真做好进城务工农民随迁子女义务教育工作，促进了农民就近就业。

折叠改革开放不断深化

政府机构改革稳步实施，警务体制、文化事业单位、集体林权制度改革扎实推进。土地保障机制不断创新，土地综合整治和节约集约用地得到加强。财政体制改革继续深化，财政管理的科学化、精细化水平进一步提高。公共资源交易体制改革成效明显，交易额突破100亿元，增收节支13亿元。金融服务和投融资平台建设得到加强。全市银行机构存款余额970.37亿元，增长22.15%;贷款余额499.4亿元，增长19.11%;利用资本市场融资29.33亿元;实现保险费收入48.24亿元，增长62.88%。加大招商引资力度，组织开展了一系列重大招商活动，成功承办了2010年全国"农洽会"。新批外商投资企业28家，合同利用外资4.16亿美元，实际利用外资1.26亿美元，增长50%。新落地市外内资项目560个，合同投资总额651亿元，增长25.1%;实际到位资金132.75亿元，增长27%。

折叠和谐社会建设扎实推进

继续加大投入，努力解决关系人民群众切身利益的问题，"十项民生工程"顺利实施。城镇居民人均可支配收入达13702元，农民人均纯收入4861元，分别增长11.3%、15.3%。就业和社会保障得到加强。全年城镇新增就业7.61万人，下岗人员再就业3.25万人，其中"4050"人员1.42万人，城镇登记失业率3.6%，农村劳动力转移就业233万人。2个县新农保试点顺利开展，企业退休人员养老金标准提高，城乡低保实现应保尽保。城镇居民医保参保总人数达到67万人，新农合参保人数达650万人。新开工廉租住房37.2万平方米、经济适用房9.8万平方米。社会事业全面发展。实施各类科技计划项目227项，开发新产品76个、新技术新工艺95项。继续加大教育投入，扎实推进中小学布局调整，共落实农村(含县城)各类义务教育经费7.55亿元，城乡就学条件进一步改善。职业教育攻坚计划顺利实施。公共文化服务体系建设扎实推进，建成了一批乡镇综合文化站、农家书屋和信息共享工程。积极开展全民健身活动，人民群众健康水平得到提高。市中心医院病房大楼等重大项目顺利实施，改造了一批乡镇卫生院、村卫生室和城市社区卫生服务中心。加强疾病预防控制和应急管理，有效控制了各类传染性疾病的发生。人口自然增长率5.29‰，稳定了低生育水平。人事、审计、统计、气象、民族宗教、外事侨务、红十字会、史志、档案、残疾人等工作都取得了新的成绩。圆满完成了对口支援四川江油灾后恢复重建工作任务。社会大局和谐稳定。认真落实各项安全生产措施，健全安全责任体系，及时排查消除各类安全隐患，安全生产形势总体平稳。加强食品药品监管，保障了人民群众的健康安全。加强社会治安综合治理，严厉打击各类刑事犯罪，命案发案数下降12.8%，现行命案侦破率达100%。积极化解各类矛盾，信访总量、赴京上访明显下降。

折叠民主法制和精神文明建设得到加强

坚持"三具两基一抓手"，深入推进"两转两提"，认真开展"创先争优"和"双整"集中活动，干部群众干事创业的氛围更加浓厚。严格依法行政，自觉接受人大及其常委会监督，支持政协参政议政，认真办理人大代表建议和政协提案。扎实推进政府系统反腐倡廉建设，积极开展政风行风评议活动。深入开展各类群众性精神文明创建活动，公民道德水平和城乡文明程度不断提升，被评为"省文明城市创建工作先进市"。高度重视驻驻部队、国防后备力量和人民防空建设，认真做好拥军优属、拥政爱民和军民共建工作，军政军民团结进一步巩固。

各位代表!2010年各项任务的完成，标志着"十一五"规划确定的主要目标顺利实现。这一时期，国际金融危机严重冲击，资源环境约束趋紧，结构性矛盾日益突出，经济社会发展形势复杂多变。面对压力和挑战，全市上下积极应对、团结拼搏、克难攻坚，取得了保增长、保民生、保稳定的重大胜利，经济社会发展迈上了新的台阶。

--这五年是经济持续快速发展、综合实力显著提升的五年。全市生产总值突破1000亿元，年均增长12.4%，较"十五"末翻了一番，人均生产总值达到13900元。全部工业增加值达到393亿元，年均增长15.1%;社会消费品零售总额年均增长18.2%;财政总收入由不足24亿元到突破60亿元，地方财政一般预算收入由12.5亿元到36.4亿元，支出由50.6亿元到172.67亿元;金融机构各项存款余额由400.41亿元到970.37亿元。

--这五年是经济结构不断优化、发展后劲明显增强的五年。食品、建材、医药、能源和煤化工、制造等支柱产业不断壮大，规模以上工业占全部工业的比重提高27个百分点;白云纸业20.4万吨文化用纸、中集华骏年产5000辆罐式车、吴桂桥煤矿等一批重点项目建成投产;文化、旅游、物流、金融等现代服务业全面提速，二、三产比重由66%提升到72.5%。万元生产总值能耗下降18%，主要污染物排放总量大幅下降。

--这五年是粮食生产能力稳步提高、"三农"工作全面加强的五年。积极筹措支农资金236亿元，投入农业农村发展，实施了一大批农田水利工程项目，新增有效灌溉面积245万亩，改造中低产田101.3万亩，农业生产条件明显改善，粮食产量连年稳定在130亿斤以上，为国家粮食安全作出了重要贡献。

--这五年是城镇化步伐全面加快、城乡面貌不断改观的五年。交通、能源等重大基础设施取得长足发展，公路通车里程达1.9万公里，发电装机容量突破200万千瓦。累计完成城镇建设投资1570亿元，城镇化率提高13.3个百分点，成功创建了省级园林城市、省级卫生城市和中国优秀旅游城市。农村水、电、路、气等基础设施和医疗卫生、文化教育等公共服务设施明显改善。

--这五年是改革开放继续深化、招商引资成效显著的五年。政府机构改革顺利实施，政府投融资体系初步形成，城信社成功组建商业银行，卫生、交通、财税等领域的改革取得新进展。累计引入市外投资项目1000多个，实际利用市外资金400多亿元。与中国昊华、中国国电、中国通用、中集集团、香港华润、中国华能、平煤集团等境内外知名企业成功实现战略合作。进出口总额年均增长30%。

--这五年是民生得到持续改善、和谐社会建设扎实推进的五年。城镇新增就业累计达35.8万人;农村劳动力转移就业达233万人，净增52万人。实现了50.46万人脱贫。社会事业长足发展，公共服务体系不断完善，社会保障体系不断健全。城镇居民人均可支配收入、农民人均纯收入年均增长14.7%和14.4%。

各位代表!"十一五"这些成就的取得，是省委、省政府和市委正确领导的结果，是市人大、市政协和社会各界积极支持的结果，也是全市人民团结奋斗的结果。在此，我代表市人民政府，向工作在全市各条战线的广大工人、农民、知识分子、干部、驻驻部队指战员、武警官兵、公安民警和各界人士致以崇高的敬意!向所有关心、支持驻马店建设和发展的同志们、朋友们表示衷心的感谢!

回顾五年来的工作，历程极不平凡，成绩来之不易。通过这些年的实践，我们进一步深化了对科学发展观的认识，提高了贯彻落实科学发展观的能力。在工作中，我们主要把握了以下几点:一是坚持发展第一要务，围绕科学发展这个主题，抢抓国家扩大内需、促进中部崛起等战略机遇，集中精力抓项目、扩总量、调结构、促转型，努力实现又好又快发展。二是坚持以提升综合竞争力、实现可持续发展为目标，强力推进产业集聚区建设，着力培育支柱产业，注重自主创新，促进产城融合，不断积蓄发展后劲。三是坚持统筹城乡、区域协调发展，积极优化区域布局，加快中心城区、县城和小城镇建设，大力发展县域经济，扎实推进新农村建设，全面加快城镇化进程。四是坚持用改革的办法破解发展难题，消除制约发展的机制体制障碍，牢固树立"市外即外"的观念，全方位、多层次、宽领域承接国内外产业转移，切实增强发展的动力和活力。五是坚持以人为本的理念，多渠道筹措资金，全面推进社会事业发展，切实解决关系人民群众切身利益的问题，多方化解社会矛盾，确保社会大局和谐稳定。六是坚持"四个重在"实践要领，深入推进"两转两提"，团结带领全市干部群众，乘势而上，克难攻坚，在复杂严峻的形势下，形成了争先恐后、竞相发展的强大气势。

在肯定成绩的同时，我们也清醒地认识到，驻马店人口多、基础差、底子薄、发展不平衡的基本市情没有根本改变。当前发展中还存在不少矛盾和问题:经济总量小、产业结构层次低、城乡结构不合理仍是我们面临的突出矛盾，加快发展方式转变显得尤为紧迫;资源环境约束日益加剧，节能减排压力加大，保持经济持续较快增长的难度增加;区域竞争日益激烈，实现平安崛起的压力增大;社会事业欠账较多，公共服务水平不高，群众上学难、看病贵等民生问题依然突出;政府工作还存在不少问题和不足，一些部门职能转变不到位，行政效能不高，少数干部工作作风不扎实，决策执行不力，领导方式需要进一步转变，发展环境亟待进一步优化。对此，我们一定高度重视，认真解决。

折叠编辑本段"十二五"发展总体思路和目标任务

市委二届九次全会通过的"十二五"规划建议，是指导驻马店今后一个时期发展的行动纲领。"十二五"时期，我市经济社会发展将呈现新的阶段性特征，经济发展进入较快增长期，经济结构进入加快调整期，公共需求进入快速扩张期。这一时期，我们既迎来产业转移、扩大内需、后发优势等难得历史机遇，也面临结构性矛盾突出、资源环境约束加剧、区域竞争压力加大、改善民生任务繁重等诸多困难。但总体上看，机遇大于挑战，有利条件多于不利因素。当前，中原经济区建设已上升为国家战略，我们必须紧紧抓住这一重大历史机遇，乘势而上，以建设豫南区域中心城市为战略定位，加快建设现代农业基地、特色产业基地和生态旅游基地，努力走出一条不以牺牲农业和粮食、生态和环境为代价的"三化"协调发展的路子，不断开创驻马店平安崛起新局面。

根据市委二届九次全会精神，"十二五"时期我市经济社会发展的指导思想是:以中国特色社会主义理论体系为指导，以科学发展为主题，以加快转变经济发展方式为主线，以全面建设小康社会、实现驻马店平安崛起为总体目标，以富民强市为中心任务，以中原经济区建设为契机，坚持重在持续、重在提升、重在统筹、重在为民的实践要领，按照"全部工作以经济建设为中心，经济建设以项目建设为重点，项目建设以工业项目为主导"的基本要求，着力实施项目带动，加快产业集聚区建设;着力调整优化结构，提升发展层次;着力加快中心城区和县城建设，推进城镇化进程;着力深化改革开放，增强发展活力;着力创新社会管理，保障改善民生，构建和谐社会，促进工业化、城镇化、农业现代化全面、快速、协调发展，形成城乡统筹、以城带乡、以工促农、资源节约、生态良好的发展新格局。

"十二五"时期经济社会发展的主要目标是:全市生产总值年均增长11%左右，按可比价格计算，2015年达到1770亿元，人均生产总值达到26000元，其中二、三产业增加值占地区生产总值的比重达到80%。城镇居民人均可支配收入和农民人均纯收入年均增长9%左右。全社会固定资产投资年均增长18%。财政一般预算收入年均增长13%左右，力争在2010年的基础上翻一番。粮食综合生产能力稳定在140亿斤以上。社会消费品零售总额年均增长17%左右。城镇化率年均提高约2个百分点。进出口总额、实际利用外资年均分别增长20%。万元生产总值能耗、二氧化碳、主要污染物排放量控制在省下达的指标内。

实现上述目标任务，在指导思想上必须把握好四个方面:一是以科学发展为主题。在新的发展时期，坚持发展是硬道理的本质要求，就是坚持科学发展，就是要把科学发展要求体现在经济社会发展的各个方面、各个环节，更加注重以人为本，更加注重全面协调可持续发展。二是以加快转变经济发展方式为主线。坚持把加快转变经济发展方式作为推动科学发展的战略任务，贯穿经济社会发展全过程和各领域，坚持在发展中促转变、在转变中谋发展。三是以实现驻马店平安崛起为总体战略。立足中原经济区建设大局，依托自身优势，遵循经济发展规律，结合实际，主动对接、主动融入，使驻马店成为中原经济区的重要经济板块。四是以富民强市为中心任务。这是推动科学发展的本质要求，是坚持以经济建设为中心在我市的具体实践。坚持富民优先、强市为要，把保障和改善民生作为各项工作的出发点和落脚点，着力加快工业化、城镇化和农业现代化进程，努力实现富民与强市的有机统一。

实现上述目标任务，在工作推进上必须突出四个重点:一是扩大投资。继续把项目建设作为重要抓手，积极论证筛选项目，千方百计争取项目，大力开展招商引资，全力抓好项目实施，努力扩大投资规模，拉动经济的快速增长。二是调整结构。突出抓好产业结构和城乡结构调整，着力解决产业竞争力弱、城乡差距大等问题，促进经济社会又好又快发展。三是改革开放。把改革创新作为破解深层次矛盾和各种瓶颈制约的根本途径，把扩大对外开放作为借势跨越、带动全局的战略举措，不断增强经济社会发展动力和活力。四是改善民生。保障和改善民生既是发展目的，又是发展动力。更加注重增加城乡中低收入居民收入，扩大社会保障覆盖面，加强社会管理创新，提升公共服务水平，促进人的全面发展。

各位代表!《驻马店市国民经济和社会发展第十二个五年规划纲要(草案)》及说明已印发各位代表，请予审议。我们坚信，有省委、省政府和市委的正确领导，有全市上下的齐心协力、奋勇拼搏，再大的困难也阻挡不了我们前进的步伐，驻马店"十二五"发展蓝图一定能够如期实现!

折叠编辑本段2011年发展目标和重点工作

2011年是实施"十二五"规划开局之年。总体上看，今年我们面临的形势依然比较复杂。一方面，国家继续加大对"三农"的投入，国内产业转移加快推进，中原经济区建设启动实施，我市的经济社会发展也呈现好的发展趋势、态势和气势，这些都有利于我们发挥优势，加快发展。另一方面，当前经济回升的不稳定、不确定因素仍然较多，国家宏观政策调整，经济发展要素供给趋紧，加之我们自身存在的突出矛盾和问题，都给我们的工作带来前所未有的压力。因此，我们既要坚定信心、抢抓机遇、加快发展，又要增强忧患意识，以更加有效的工作，确保"十二五"开好局、起好步。

根据市委经济工作会议总体要求，今年经济社会发展的主要预期目标是:生产总值增长11%以上，规模以上工业增加值增长18%，全社会固定资产投资增长18%，城镇固定资产投资增长20%，社会消费品零售总额增长17%，地方财政一般预算收入增长13%，人口自然增长率控制在6.5‰以内，居民消费价格涨幅控制在4%左右，城乡居民收入较快增长，节能减排完成省定目标。

围绕上述要求和目标，做好今年政府工作，必须始终把发展作为第一要务，坚持"四个结合"，努力实现"四个提升"。坚持发展与转变相结合，努力提升质量和效益。把加快发展作为解决我市经济社会发展主要矛盾和突出问题的根本途经，切实做到在发展中调整结构、在发展中转变方式、在发展中提升质量、在发展中增加效益。坚持存量调整与增量带动相结合，努力提升优化经济结构。着力提高二、三产业和规模以上工业、高新技术产业、民营经济、外向型经济、财政收入等六个比重。坚持强动力与增活力相结合，努力提升招商引资水平。加大改革开放力度，着力破解"资金、土地、人才"三大要素制约，积极承接产业转移。坚持经济发展与社会发展相结合，努力提升城乡居民收入和消费水平。落实好就业、社保、教育、卫生、文化等惠民措施，切实做到在发展中改善民生、在改善民生中凝聚力量，促进经济平稳较快发展与社会和谐稳定。在具体工作中，重点抓好以下八个方面的工作:

折叠着力扩大市场需求，保持经济平稳较快发展

需求不足是制约我市经济发展的突出问题，必须通过扩大需求规模、优化需求结构，保持经济平稳较快发展。着力扩大投资需求。积极实施"1261"重点项目推进计划，重点围绕粮食核心区、产业集聚区、城镇基础设施建设等领域，组织实施10个总投资10亿元以上的项目、20个总投资5亿元以上的项目、60个总投资1亿元以上的项目和"十大民生工程"，确保项目总投资规模达到1000亿元，带动城镇固定资产投资突破600亿元。今年初步安排重点建设项目111个，总投资485.6亿元。其中，工业项目72个，总投资338.4亿元。对于这些项目，要继续加大服务协调力度，切实解决资金、用地需求，推动项目顺利实施，确保投资稳定增长。努力扩大消费需求。落实国家调整收入分配的各项政策，着力提高城乡低收入群体收入，扩大中等收入群体，增强居民消费能力。认真落实家电、摩托车下乡和家电以旧换新政策，加快"万村千乡"和"双百"工程建设。积极发展文化旅游、休闲健身消费，促进消费结构优化升级。大力整顿和规范市场秩序，营造良好消费环境。积极扩大出口。把承接产业转移作为带动出口的主要途径，努力引进一批出口带动能力强的大企业和大项目。落实鼓励出口的政策措施，重点支持优质农畜产品、医药、化工、帽服等传统产品的出口，逐步提高机电产品、高新技术产品出口规模。继续搞好企业服务。完善企业服务长效机制，着力为企业办实事、解难题。积极引导市内企业强化市场联合，互为市场解决销路问题，不断提升企业的抗风险能力。继续组织多种形式的银企洽谈会、项目推介会，加强银企对接，扩大信贷规模，不断强化金融支持。加强经济运行监测，做好煤、电、油、气、运协调工作，保障要素供给。

折叠加快产业结构调整，促进经济更好发展

以产业集聚区为载体，以转型升级项目建设为抓手，着力培育优势产业和优势企业，促进产业结构转型升级。推进工业结构优化升级。坚持以技术创新、管理创新、制度创新改造提升传统产业，积极培育生物医药、精细化工、新能源、新材料、电子信息等现代新兴产业，引领带动全市产业结构升级。引导资金、技术、人才等要素资源向优势产业集中，加快医药、能源和煤化工、食品、制造、建材基地建设，不断壮大产业集群。继续支持重点企业实施联合重组，着力培育一批大企业和企业集团。加快产业集聚区建设。按照"四集一转"要求，创新产业集聚区管理体制和投入机制，完善园区水、电、路、管网、通信等配套设施，建好投融资中心、中小企业担保中心、土地开发整理中心等服务平台，大力承接产业转移，引导老城区企业"退二进三、入园进区"，培育壮大一批产业集群和产业基地，实现产业聚集和城市发展的互相促进。大力发展现代服务业。积极推动金融保险、现代物流、信息咨询、文化旅游等现代服务业扩张规模、优化结构、提升水平。以发展壮大金融机构、金融市场为主体，进一步完善金融服务体系，积极引进国内金融保险机构。认真抓好企业上市工作，扩大直接融资规模。大力发展农产品、食品等行业物流，加快豫南农产品交易中心建设，支持天元粮油配送、大程东方粮食物流等重点项目建设。充分挖掘整合文化资源，积极培育文化企业，促进文化产业发展。继续推进旅游资源整合，坚持以嵖岈山旅游集聚区为龙头，加快建设金顶山生态旅游区、竹沟红色旅游区，加大南海禅寺、铜山、白云山等景区开发力度，推动薄山湖管理体制改革，努力打造旅游精品线路和精品景区。加强自主创新能力建设。积极筹建科技创新公共服务平台，大力培育和引进科技人才，搞好科研攻关，努力开发一批具有自主知识产权的新产品、新技术。鼓励天方药业、昊华骏化等技术研发中心，承接国家、省级研发任务;支持中集华骏铸造、泌阳夏南牛科技公司等企业建成省级工程技术研究中心。大力实施质量兴市战略，提高产品质量安全水平。扎实推进节能减排和土地集约节约利用。坚决落实节能减排目标责任制，严格执行产业政策，继续淘汰落后产能，突出抓好高耗能行业和重点领域的节能降耗。加强重点流域、区域、行业污染综合整治和农村环保工作，抓好污水、垃圾处理设施建设和运营，确保化学需氧量、二氧化硫、氨氮、氮氧化物减排达标。实施节能科技创新工程，大力开发风能、太阳能、生物质能等可再生能源。坚持最严格的耕地保护和节约集约用地制度，建立健全土地整治、节约集约用地和监督管理机制，稳妥推进城乡建设用地增减挂钩，保障发展用地需要。加大土地执法监察力度，严厉查处违法违规用地行为。

折叠大力发展现代农业，推进农村改革发展

以中原经济区建设为动力，进一步强化"三农"工作，为工业化、城镇化和农业现代化协调发展打下坚实基础。全面落实强农惠农政策，加快发展现代农业，努力把我市建成河南重要的农产品加工基地和豫南重要的农产品物流中心。认真落实强农惠农政策。继续加大对"三农"的投入，建立健全产粮大县利益补偿机制，认真执行国家稳步提高重要粮食品种最低收购价政策，引导金融机构加大涉农信贷投放，积极开展农村信用合作，做好政策性农业保险工作，调动农民生产积极性。巩固提高粮食综合生产能力。以粮食生产核心区建设为契机，重点抓好一批大中型灌区续建配套、中低产田改造和高标准农田示范工程建设;加快农业综合开发重点县、产粮大县以及县区土地治理等项目建设，确保新增和恢复有效灌溉面积40万亩、旱保田30万亩，改造中低产田15万亩，粮食总产稳定在130亿斤以上。积极推进农业结构调整。紧紧围绕蔬菜、花木、花生、食用菌、铜山优质烟等农产品，因地制宜发展特色农业、品牌农业。加快现代畜牧业发展，继续抓好生猪调出大县、肉牛发展大县建设，改造建设一批标准化生猪、奶牛和夏南牛规模养殖场。扎实推进农业产业化，着力培育壮大一批规模较大的龙头企业和生产基地。继续改善农村生产生活条件。加快农村安全饮水、道路、供电、沼气等基础设施建设，大力推进农业机械化，力争再解决40万农村人口的安全饮水问题，新增农村沼气用户1.5万户，完成100个贫困村的整村推进任务，解决脱贫人口10万人。深化农村体制改革。健全农村土地承包经营权流转机制，完善土地流转服务体系。深化集体林权制度、县级供销社体制改革。全面实施村级公益事业建设"一事一议"财政奖补政策。建立健全乡镇或区域性农业公共服务机构。

折叠统筹城乡发展，加快推进城镇化进程

坚持以重点基础设施建设为抓手，以推进"三区两县一体化"为重点，全面实施城乡建设三年行动计划，促进城乡互动、良性发展。积极推进"三区两县一体化"建设。高水平编制200平方公里新一轮城市总体规划和各专项规划，抓好高铁客运站附近地区控规和修规编制，尽快打通驿城大道，推进"工业长廊"建设，努力形成带状城市格局，构筑"三区两县一体化"城镇发展体系，力争中心城区建成区面积达到70平方公里，人口达到70万人。加强城市建设和管理。加快中心城区基础设施建设，重点抓好"四河"(练江河、骏马河、冷水河、小清河)治理和"四路"(驿城大道、汝河大道、铜山大道、开源大道)改造，实施驻马店火车站升级改造和高铁客运站场建设，推进市第二污水厂和市第一污水处理厂配套再生水厂建设。大力发展城市公交，切实解决城市交通拥堵等问题。深入开展"四城联创"活动，加强环境综合整治，加大旧城改造力度，积极实施城中村、城乡接合部村庄改造，继续实施绿化美化亮化工程，努力改善人居环境。加快县城和重点镇建设，完善基础设施和公共服务设施，增强城镇吸纳集聚能力。创新城市发展体制机制。全面推进城镇建设投融资体制改革，鼓励和引导金融机构、民间资本参与建设，积极吸引更多的外商投资城建项目。加快推进城镇户籍制度改革，解决好进城务工人员的安居、子女就学、社会保障等问题，促进农村人口有序转移。扎实推进新农村建设。坚持规划先行、就业为本、群众自愿、量力而行原则，稳步推进新农村建设。在产业集聚区、城市新区和近郊区，加快推进新型农村社区建设，率先实现城乡一体化。不宜居住的村庄以及偏远山区零散村庄，创造条件建设集中居住区，逐步实施整村搬迁。其他广大农村按照城乡统筹发展要求，逐步提高公共服务水平，改善生产生活条件。

折叠加快基础设施建设，增强加快发展支撑能力

切实加强水利、交通、能源、信息化等基础设施建设，提升服务功能，培育竞争优势，积蓄发展后劲。加强水利设施建设。抓住国家加大水利投资的机遇，多渠道增加投入，加快小型农田水利重点县建设，推进小洪河、汝河等中小河流治理，实施好薄山、板桥、宿鸭湖三大灌区续建配套和节水改造项目，抓好中小病险水库除险加固及杨庄滞洪区安全建设，开工建设蛟停湖滞洪区安全项目。加强交通体系建设。加快化新高速、石武客运专线建设进度，确保年底前建成通车;抓好京港澳高速驻马店段改扩建、市区北连接线和连接上蔡、正阳快速通道建设，确保完成年度建设任务。继续推进干线公路大中修和农村公路建设。加强能源基地建设。加快国电驻马店热电供热机组建设进度，尽快实现"双投"。开工建设华润安里煤矿、泌阳风电。积极推进城乡智能电网发展，加快中心城区、产业集聚区配套电网建设;实施一批新能源和可再生能源项目建设。加强信息网络建设。以数字化建设为重点，加快推进"三网"融合和3G网络建设，提高信号覆盖面;推进城市有线电视数字化整体转换和双向改造。加强生态体系建设。深入开展"林业生态乡镇"和"全国绿化模范县"创建活动，继续搞好山区造林和平原绿化，确保完成造林23.65万亩、森林抚育12万亩，力争全市60%的平原乡镇、40%的山区丘陵区建成林业生态乡镇，确保成功创建林业生态市。

折叠深入推进改革开放，破解经济社会发展难题

坚持把改革开放作为带动全局的战略举措来抓，不断增强发展的动力和活力。全面深化各项改革。继续深化国有企业改革，加强与央企、省企的战略合作。全面推进商贸、建筑、交通等领域的改革。加快推进市直机关所属企业脱钩改制工作。推进经营性文化事业单位改革，完成市、县影剧院、电影公司转企改制，全面实现市、县文化市场综合执法。加快医药卫生体制改革，积极实施国家基本药物制度，完成三年五项重点改革任务。不断深化金融改革，积极推进教育体制改革。继续开展大招商活动。依托产业集聚区，建设一批承接产业转移的示范园区，吸引沿海地区食品、纺织服装、鞋业、中小型电子等劳动密集型产业向我市转移。继续做好今年全国"农洽会"的筹办工作，力求取得更大的成效。不断改进和创新招商方式，重点围绕支柱产业、骨干企业、重大产业基地建设和发展，瞄准国内外知名企业，大力开展定向招商，切实增强招商引资的针对性和实效性。加强对已签约项目的跟踪问效，切实提高项目履约率、资金到位率。强化招商引资责任，严格考核，兑现奖惩，努力实现招商引资工作新突破。切实抓好利用外资工作。继续加强与国(境)外企业的交流与合作，吸引更多的境外资金进入现代服务业、节能环保和现代农业，参与我市企业重组和传统产业技术改造，努力提高利用外资水平。

折叠加强社会建设，切实保障和改善民生

继续实施好"十大民生工程"，创新社会管理，让发展成果惠及广大人民群众。着力扩大就业。实施更加积极的就业政策，大力发展劳动密集型产业、服务业，扶持自主创业和自谋职业。加强就业技能培训、就业信息服务和就业援助，重点解决高校毕业生、农村转移劳动力、退役军人的就业问题，确保全年城镇新增就业人员4.2万人，实现下岗失业人员再就业1.8万人，解决"4050"人员就业0.8万人，新增农村劳动力转移就业10万人。加强社会保障工作。继续扩大养老保险覆盖面，落实基本养老保险关系跨地区转移接续办法。落实企业退休人员基本养老金、城乡居民低保、农村五保户供养和优抚补助对象正常调整机制。扎实做好新农保试点工作。完善城镇职工、居民医疗保险制度和新农合制度，全面实现城镇基本医疗保险即时结算和市级统筹，实现新农合跨区域即时结报。切实解决失地农民生活保障问题，实现被征地农民社会保障制度全覆盖。大力发展残疾人事业，切实抓好0至6岁残疾儿童抢救性康复项目实施。加大保障性安居工程建设力度，加快棚户区和农村危房改造，开工建设各类保障性住房62万平方米。大力发展社会事业。完善农村义务教育经费保障机制。制定实施学前教育三年发展规划，逐步缓解入园难问题。启动新一轮农村中小学布局调整，加快中心城区、县城中小学规划和建设，新扩改建中心城区8所中小学。整合职教资源，推进职教园区建设。加快发展高等教育，启动驻马店幼儿师专和驻马店医专创建工作。全面完成市博物馆、群艺馆和广电中心建设，抓好市体育馆、体育中心、市直老干部活动中心建设，启动报业大厦筹建工作，抓好乡镇综合文化站、农家书屋和信息共享工程等文化项目实施。抓好市中心医院病房大楼和北区分院等项目建设，继续实施县级医院及社区卫生服务站、乡镇卫生院、村卫生室建设改造。切实抓好艾滋病等重大传染病的防治工作。广泛开展群众性健身运动，努力提高全民身体素质。继续做好人口计划生育工作，稳定低生育水平。继续做好人事、审计、统计、外事侨务、红十字会、档案、史志、地震、气象等工作。加强市场物价监管。落实价格监管各项措施，坚决打击囤积居奇、哄抬价格等不法行为。建立和完善最低生活保障、失业保险标准与物价上涨挂钩的联动机制，保障低收入群体基本生活。努力提高全民文明素质。大力弘扬艰苦奋斗、求真务实精神，积极推进社会主义核心价值体系和社会公德、职业道德、家庭美德、个人品德建设，深化大学生思想政治教育和未成年人思想道德建设。深入开展"扫黄打非"，扎实做好网络管理，营造良好社会文化环境。深入开展群众性精神文明创建活动，提高城乡文明程度。全力维护生产安全和社会稳定。全面启动"安全驻马店"创建活动，认真落实安全生产责任制，加强重点领域隐患排查整治，健全安全生产投入保障机制，防止重特大事故发生。加强食品药品安全监管。深入开展平安建设，推进社会矛盾化解、社会管理创新、公正廉洁执法三项重点工作，坚决纠正损害群众利益的突出问题，落实重点项目稳定风险评估机制，妥善处理土地征用、拆迁安置、涉法涉诉等方面的矛盾，保障群众合法权益。加强信访工作，积极预防和妥善处置群体性事件和突发公共事件。继续加强社会治安综合治理，健全社会防控体系，依法防范和严厉打击各类违法犯罪活动。全面贯彻党的民族宗教政策，切实做好民族宗教工作。加强国防教育和国防后备力量建设，支持驻驻解放军、武警部队、预备役部队、民兵和人民防空建设。深入开展双拥共建，争创"全国双拥模范城"，巩固发展军政军民团结。

折叠切实加强政府自身建设，努力提高施政水平

做好今年各项工作，必须以领导方式转变加快发展方式转变，坚持"三具两基一抓手"，深入推进"两转两提"，扎实开展"创先争优"活动，努力建设人民满意的政府。坚持依法行政。严格执行人大及其常委会的决议、决定，自觉接受人大法律监督和政协民主监督，认真办理人大代表建议和政协委员提案，充分听取各民主党派、工商联、无党派人士及各人民团体的意见，强化监察、审计等专门监督，广泛接受社会公众和新闻舆论的监督。支持工会、共青团、妇联等人民团体发挥桥梁纽带作用。规范行政执法行为，落实行政执法责任和过错追究制度，提高行政执法水平。坚持依法决策、科学决策、民主决策，增强工作的前瞻性、系统性。深入推进政务公开，重点推进财政预算、公共资源配置、重大建设项目审批等领域的信息公开，接受社会监督。注重求实求效。强化机遇意识、责任意识，做到责随职走、心随责走，求真务实、真抓实干。突出运作，狠抓落实，说到做到、说好做好，提高政府执行力和公信力。深入基层和群众，搞好调查研究，多到一线和现场去解决问题。精简会议、文件，减少不必要应酬和检查评比，腾出更多的时间和精力谋大事、抓落实、搞服务、促发展，努力创造出经得起历史和人民检验的业绩。提高服务效能。深化行政管理体制改革，减少和规范行政审批。加强和规范行政服务中心、公共资源交易中心建设，认真落实联审联批、限时办结制等制度。深入开展政风行风评议，加强监督考评，强化行政问责，重点整治行政不作为、乱作为、慢作为等行为，努力提高服务质量和行政效率。加强廉政建设。牢固树立宗旨意识，坚持以人为本、执政为民，恪守为民之责，多办利民之事，真心实意为群众排忧解难。大力倡导勤俭节约、艰苦朴素之风，坚决反对挥霍公款和铺张浪费。认真落实党风廉政建设责任制，注重从源头上预防和治理腐败，坚决查处违纪违法案件，以党风廉政建设和反腐败斗争实效取信于民。

各位代表!承继"十一五"成就，谱写"十二五"新篇，是时代赋予我们的光荣使命和神圣职责。让我们在省委、省政府和市委的坚强领导下，深入贯彻落实科学发展观，解放思想，坚定信心，克难攻坚，扎实工作，凝聚全市人民的智慧和力量，努力开创我市经济社会发展的新局面，以优异成绩迎接中国共产党成立90周年!
